# MicroRNAs mediated regulation of MAPK signaling pathways in chronic myeloid leukemia

**DOI:** 10.18632/oncotarget.7977

**Published:** 2016-03-08

**Authors:** Chiranjib Chakraborty, Ashish Ranjan Sharma, Bidhan Chandra Patra, Manojit Bhattacharya, Garima Sharma, Sang-Soo Lee

**Affiliations:** ^1^ Institute for Skeletal Aging & Orthopedic Surgery, Hallym University-Chuncheon Sacred Heart Hospital, Chuncheon, 200704, Korea; ^2^ Department of Bio-informatics, School of Computer and Information sciences, Galgotias University, Greater Noida, Uttar Pradesh, 203201, India; ^3^ Aquaculture Research Unit, Department of Zoology, Vidyasagar University, Midnapore, West Bengal, 721102, India

**Keywords:** chronic myeloid leukemia, microRNA, signaling pathway, oncogenic, MAPK

## Abstract

Chronic myeloid leukemia (CML) is a severe problem throughout the world and requires identification of novel targets for its treatment. This multifactorial disease accounts for about 15% of the all diagnosed leukemia cases. Mitogen-activated protein kinase (MAPK) signaling pathway is crucial for the cell survival and its dysregulation is being implicated in various types of cancers. In here, we have discussed the potential role of various miRNAs that are found involved in regulating the proteins cascades of MAPK signaling pathway associated with CML. An emphasis has been paid to summarize the influence of various miRNAs in elevating or suppressing the expression level of significant proteins such as miR-203, miR-196a, miR-196b, miR-30a, miR-29b, miR-138 in BCR-ABL tyrosine kinase; miR-126, miR-221, miR-128, miR-15a, miR-188-5p, miR-17 in CRK family proteins; miR-155, miR-181a with SOS proteins; miR-155, miR-19a, with KRAS proteins; miR-19a with RAF1 protein; and miR-17, miR-19a, miR-17-92 cluster with MAPK/ERK proteins. In light of ever-increasing importance and ever-widening regulatory roles of miRNAs in cells, we have reviewed the recent progress in the field of miRNAs and have tried to suggest them as controlling targets for various protein cascades of MAPK signaling pathway. An understanding of the supervisory mechanism of MAPK by miRNAs might provide novel targets for treating CML.

## INTRODUCTION

Among evolutionarily conserved pathways, mitogen-activated protein kinase (MAPK) pathways link extracellular signals to their intracellular targets and controls fundamental cellular processes such as cell proliferation, cell growth, cell migration, cell differentiation, embryogenesis and cell death [1–3]. The first MAP kinases were identified in the pheromone pathway of the budding yeast (*Saccharomyces cerevisiae*), Kss1p and Fus3p in between 1989 and 1991 [4]. Till date, 14 MAPKs have been recognized in mammals belonging to seven groups. These seven groups may further be classified into two broad categories, either conventional or atypical MAPKs [5]. Conventional MAPKs includes extracellular signal-regulated kinase (ERK)1/2, ERK5, Jun N-terminal kinase (JNK)1/2/3, p38 isoforms α/β/γ(ERK6)/δ while atypical MAPKs comprises of ERK3/4, ERK7 and Nemo like Kinases (NLK). Since, MAPK pathways are crucial for the every aspect of cell survival and growth, any irregularities may impose cancerous properties to cells, like independence from proliferation signals, infinite replicative potential, capability to invade and metastasize, attract and endure angiogenesis for nutrient supply, evasion of apoptosis, attainment of drug resistance and evasion of oncogene induced senescence. To illustrate the significance of MAPK in cancers, a number of reviews have highlighted their role in various types of cancers [1, 6–8].

Chronic myeloid leukemia (CML) is a rare clonal myeloproliferative malignancy of pluripotent hematopoietic stem cells [9, 10]. It was perhaps the first form of leukemia to be acknowledged and may account for up to 15% of reported cases of leukemia in the developed world [11], though global prevalence is not known. CML is caused due to a reciprocal translocation between chromosomes 9 and 22 t(9;22) (q34;q11) which generate an abnormal fusion gene, *BCR-ABL*. Gene product of *BCR-ABL* exerts a constitutive tyrosine kinase activity crucial for the function of several signaling pathways involved in various malignancies, including CML [12]. Accordingly, a modifications in most of the members of the MAPKs have been observed due to BCR/ABL transformation and have been found associated with cell survival or drug resistance [8, 13, 14]. Taking into consideration the importance of MAPK in CML, specific inhibitors for BCR-ABL tyrosine kinase activity have been designed and are being developed for treating CML [15].

microRNAs (miRNAs) consist of a large family of short (~22-nucleotides in length) noncoding RNAs [16, 17] that are not translated into proteins and control target gene expression in metazoan animals, plants, and protozoa, especially through post-transcriptional and translational regulation [18]. miRNAs regulate gene expression by cleaving the target mRNAs directly or inhibiting translation through perfect or nearly perfect complementary base pairing to targeted mRNAs at the 3′ untranslated regions (UTRs) [19–23]. This class of RNA was initially discovered in 1993 by Ambros and colleagues, who described a 22-nucleotides RNA in *Caenorhabditis elegans* encoded by the lin-4 gene [24]. However, miRNAs that might be regulating various crucial cellular functions and pathways of a given cell are yet be revealed completely. Recent studies on miRNAs associated with human diseases have indicated that these tiny molecules play a crucial role in controlling cellular signal transduction cascades. The expression profile of miRNAs in CML was first studied by Zhu et al., describing that the regulatory mechanism of miRNAs can regulate the expression of several CML targets [25]. From then, several miRNAs have been identified and are known to be associated with the signaling cascade pathways related to CML.

As the role of various miRNAs are being identified in the pathogenesis of CML and MAPK signaling pathways is known to play a crucial role in the development of CML, in this review, we have tried to discuss the regulatory role of miRNAs in MAPK signaling cascade. Particular attention has been paid to explain that how the expression pattern of the small miRNAs might regulate the components of MAPK signaling pathway related to pathogenesis of CML. For this, we have highlighted the miRNA mediated regulation of different proteins involved in MAPK signaling cascade, such as BCR-ABL, CRK, CRKL, KRAS, RAF1, as well as MAPK1. An understanding of the expression pattern and regulative role of miRNAs in controlling the components of MAPK signaling pathway might help to design the approaches required to combat CML.

### miRNAs and MAPK signaling pathway in CML

**Figure 1:**
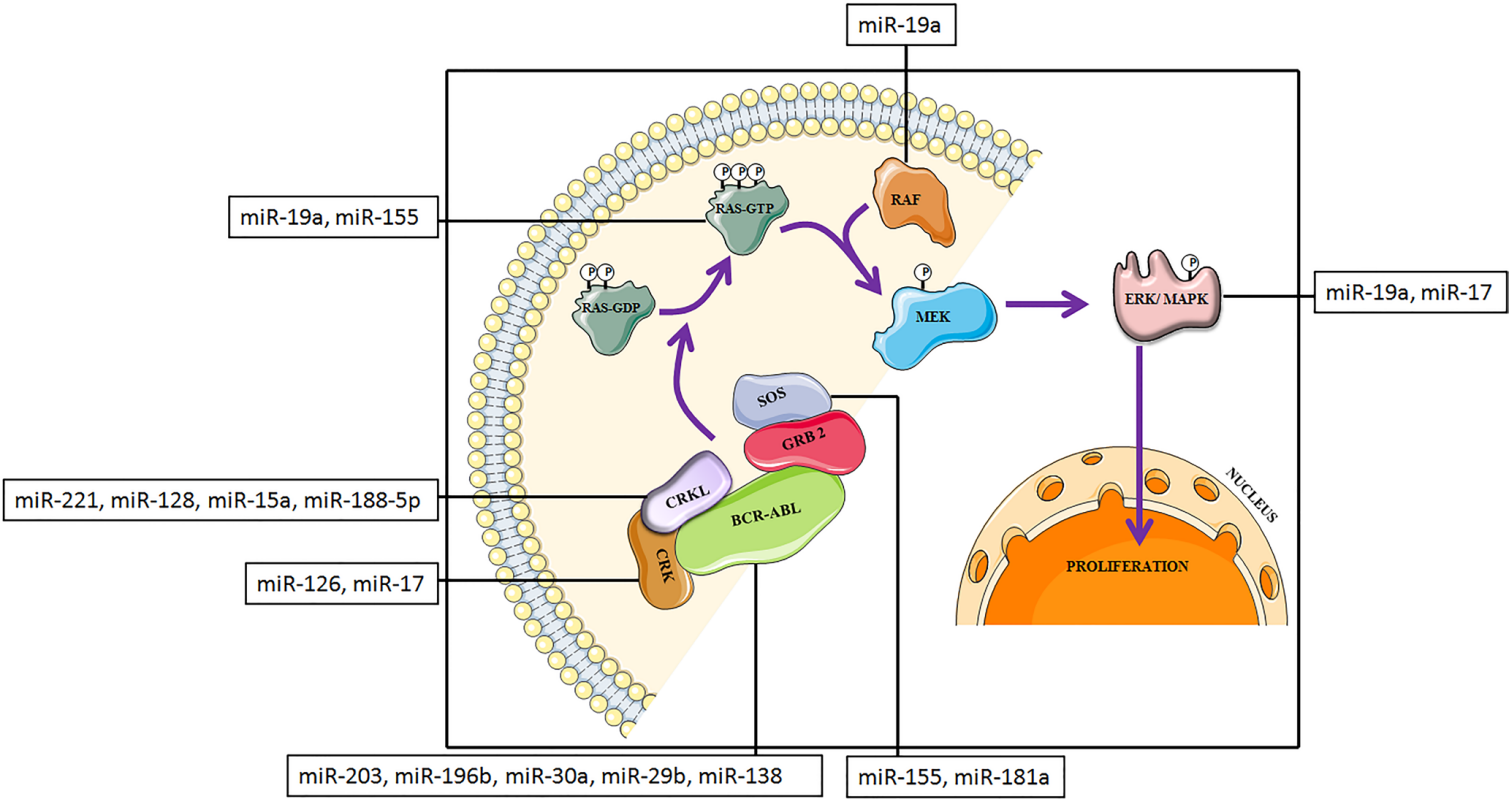
Schematic representation of MAPK protein cascade downstream of BCR-ABL transformed cells with miRNAs that target MAPK signaling pathway components

CML involves an extremely complex network of signaling cascade mechanism. Cytogenetically, it is characterized by the presence of the Philadelphia chromosome (Ph), which originates from the reciprocal translocation between chromosome 9 and 22 [26–30]. Approximately 90% of patients with CML have this acquired genetic abnormality [28]. Due to translocation, the *BCR* gene from chromosome 22 is fused to the *ABL* gene on chromosome 9 which generate an abnormal *BCR-ABL* fusion gene. This fusion gene encodes a fusion protein with tyrosine kinase activity and transforming ability which activates downstream signal transduction pathways involved in CML [26]. It is well known that the MAPK pathway is an important downstream signaling cascade in several types of cancer [31] as well as various other cellular mechanisms. The MAPK signaling cascade is a highly conserved component and plays a central role in CML (Figure 1). This pathway is necessary for the transcription of genes involved in cell proliferation and survival [32, 33]. In CML, auto-phosphorylation of tyrosine 177 on BCR-ABL fusion protein provides a docking site for the adapter molecule, growth factor receptor-bound protein 2 (GRB-2) [34]. Subsequently, GRB-2, binds to the SOS protein, which stabilizes a GTPase, RAS, in its dynamic GTP-bound form. Other two adapter molecules, Src homology 2 domain containing (SHC), and CRKL, can also activate RAS. Both acts as substrates for BCR-ABL [35, 36] and bind BCR-ABL through their Src Homology (SH) 2 (SHC) or SH3 (CRKL) domains. Activated RAS prompts the kinase activity of RAF. Activated RAF in turn initiates a signaling cascade through the serine–threonine kinases MEK1/MEK2 and ERK (MAPK), which eventually leads to the activation of gene transcription [37].

In these circumstances, miRNAs may regulate the expression of proteins at different levels by binding with the complementary sequence of 3′ untranslated region of the target mRNAs, thereby controlling the signal transduction processes [38]. Several miRNAs play significant role in the MAPK signaling pathway and among them, the following miR-203, miR-196b, miR-29b, miR-30a, miR-138, miR-155, miR-19a, miR-17, miR-126, miR-128, miR-221, miR-15a, miR-188-5p and miR-181a are well studied (Table 1, 2) [39].

**Table 1:** Different Human miRNA and Their Chromosomal Location, Gene Location, pre-miRNA Length, Mature Sequence Associated with the MAPK signaling pathway in CML

Name of miRNA	Chromosomal (Ch) Location	Gene location (EXON/INTRON/UTR)	Pre-miRNA length (nucleotide; nt)	Mature Sequence
miR-203	Ch14q32.33	intergenic	110 nt	65| 5′- GUGAAAUGUUUAGGACCACUAG -3′ |86
miR-196b	Ch7 p15.2	3UTR + 3/ intron + 1/ exon + 1/ intron + 1/ exon −1/ exon −2	84 nt	15| 5′- UAGGUAGUUUCCUGUUGUUGG -3′ |35
miR-29b-1	Ch7 q32.3	Intergenic	81 nt	51| 5′- UAGCACCAUUUGAAAUCAGUGUU -3′ |73
miR-29b-2	Ch1 q32.2	intergenic	81 nt	52| 5′- UAGCACCAUUUGAAAUCAGUGUU -3′ |74
miR-30a	Ch6 q13	intron + 3/ intron + 3	71 nt	6| 5′- UGUAAACAUCCUCGACUGGAAG -3′ |27
miR-138-1	Ch3p21.32	intergenic	99 nt	23| 5′- AGCUGGUGUUGUGAAUC -3′ |39
miR-138-2	Ch16q13	intergenic	84 nt	10| 5′- AGCUGGUGUUGUGAAUC -3′ |26
miR-155	Ch21 q21.3	intergenic	65 nt	4| 5′- UUAAUGCUAAUCGUGAUAGGGG -3′ |25
miR-19a	Ch13 q31.3	3UTR + 2/ intron + 3/ intron + 2/ intron + 3	82 nt	49| 5′- UGUGCAAAUCUAUGCAAAACUGA -3′ |71
miR-17	Ch13 q31.3	3UTR + 2/ intron + 3/ intron + 2/ intron + 3	84 nt	14| 5′- CAAAGUGCUUACAGUGCAGGUAGU -3′ |37
miR-221	ChX p11.3	intergenic	110 nt	65| 5′- AGCUACAUUGUCUGCUGGGUUUC -3′ |87
miR-181a-1	Ch1 q32.1	intergenic	110 nt	24| 5′- AACAUUCAACGCUGUCGGUGAGU -3′ |46
miR-181a-2	Ch9 q33.3	intron −2/ intron −2/ intron −2/ intron + 1	110 nt	39| 5′- AACAUUCAACGCUGUCGGUGAGU -3′ |61
miR-126	Ch9q34.3	Intron +7/ Intron +7/ Intron +6/ Intron +6/ Intron +5/ Intron +7/ Intron +7	85 nt	15| 5′- CAUUAUUACUUUUGGUACGCG -3′ |35
miR-128a	Ch2q21.3	Intron +15/ Intron +18	82 nt	50| 5′- UCACAGUGAACCGGUCUCUUUU -3′ |71
miR-128b	Ch3p22.3	Intron +17/ Intron +18/ Intron +18/ Intron +11/ Intron +17	84 nt	52| 5′- UCACAGUGAACCGGUCUCUUUC -3′ |73
miR-15a	Ch13q14.2	intron + 4/ intron + 4/ intron + 5/ intron + 5/ intron + 4/ intron + 3/ intron + 4/ intron + 3	83 nt	14| 5′- UAGCAGCACAUAAUGGUUUGUG -3′ |35
miR-188	ChXp11.23	intron + 3/ intron + 3/ intron + 3/ intron + 3/ intron + 3	86 nt	15| 5′- CAUCCCUUGCAUGGUGGAGGGU -3′ |36

**Table 2:** Various human miRNA and their synonym, miRNA Map accession no. and HGNC ID associated with MAPK signaling pathway in CML

Name of miRNA	miRNAs synonym	miRNA Map accession no.	HGNC [HUGO gene nomenclature committee] ID
miR-203	hsa-miR-203	MI0000283	HGNC:31581
miR-196b	hsa-miR-196b	MI0001150	HGNC:31790
miR-29b-1	hsa-miR-29b-1	MI0000105	HGNC:31619
miR-29b-2	hsa-miR-29b-2	MI0000107	HGNC:31620
miR-30a	hsa-miR-30a	MI0000088	HGNC:31624
miR-138-1	hsa-miR-138-1	MI0000476	HGNC:31524
miR-138-2	hsa-miR-138-2	MI0000455	HGNC:31525
miR-155	hsa-miR-155	MI0000681	HGNC:31542
miR-19a	hsa-miR-19a	MI0000073	HGNC:31574
miR-17	hsa-miR-17	MI0000071	HGNC:31547
miR-221	hsa-miR-221	MI0000298	HGNC:31601
miR-181a-1	hsa-miR-181a-1	MI0000289	HGNC:31590
miR-181a-2	hsa-miR-181a-2	MI0000269	HGNC:31549
miR-126	hsa-miR-126	MI0000471	HGNC:31508
miR-128a	hsa-miR-128-1, hsa-miR-128a	MI0000447	HGNC:31510
miR-128b	hsa-miR-128-2, hsa-miR-128b	MI0000727	HGNC:31511
miR-15a	hsa-miR-15a	MI0000069	HGNC:31543
miR-188	hsa-miR-188	MI0000484	HGNC:31559

### miRNAs and cascades of MAPK signaling

#### BCR-ABL fusion protein

It is well recognized that BCR-ABL tyrosine kinase activity plays a crucial role in CML [40]. Most of the patients with CML possess breakpoints in intron 1 or 2 of the *ABL* gene and in the main breakpoint cluster region (M-bcr) of the *BCR* gene, either amongst exons 13 and 14 (b2), or else 14 and 15 (b3) [41]. These breakpoints produce *BCR/ABL* fusion genes that transcribe either a b2a2 or b3a2 mRNA. The final product of this gene reorganization is a 210 kDa cytoplasmic fusion protein, p210BCR/ABL. This fusion protein is vital and adequate for the malignant transformation of CML, and is liable for the phenotypic anomalies of chronic phase CML [42–44]. Several functional domains have been established in the BCR-ABL protein that may contribute to cellular transformation. In the ABL portion, these domains are; SH1 (tyrosine kinase), SH2, and actin-binding domains. Whereas in BCR portion, there are coiled-coil oligomerization domain (amino acids (aa) 1–63), GRB-2 binding site (tyrosine at location 177) and the phosphoserine/threonine rich SH2 binding domain [41, 45].

A number of miRNAs regulate the expression of BCR-ABL, of which, miR-203 has been extensively studied. It essentially regulates ABL1 and BCR-ABL1 levels and inhibit cell proliferation [39, 46]. miR-203 is located intergenically on human chromosome 14 (Ch14q32.33) [47] and has been identified as a skin-specific miRNA. In normal condition it promotes epidermal differentiation by inducing cell-cycle exit and restricting proliferative potential [48]. It is exclusively expressed in keratinocytes (most common cell type in the epidermis), but not in the hair follicles of the skin. It has been demonstrated that over expression of miR-203 reduces ABL1 and BCR-ABL1 fusion protein levels in an ABL1-dependent manner [46]. A recent study of combined treatment effects of miR-203 and imatinib (small molecule kinase inhibitor) on imatinib-resistant cell lines demonstrated that miR-203 can serve as a novel target for CML treatment [49]. Another study also revealed that imatinib provoke the demethylation of the miR-203 promoter region, resulting in low expression of targeted *BCR-ABL1* genes, and loss of proliferation of leukemic cells [50]. After noticing that both, the murine and the human, 3′ UTR of *ABL1* genes contain miR-203 target sequences and this target site is well conserved in other vertebrates, Bueno et al. suggested that miR-203 may control ABL1 levels in a variety of organisms [46]. It was further suggested that miR-203 functions as a tumor-suppressor miRNA, targeting BCR-ABL and ABL kinases, which is epigenetically silenced in human Ph-positive leukemia cell lines [51, 52].

miRNA-196b is a vertebrate-specific miRNA, which appears to be expressed from intergenic regions in *Homobox* (*HOX)* gene clusters in many vertebrate species [53]. It belongs to the miR-196 family. Three miR-196 genes have been found so far i.e., miR-196a-1, miR-196a-2 and miR-196b [54]. In humans, the gene for miR-196b is located in a highly evolutionarily conserved region between *HOXA9* and *HOXA10* genes, on chromosome 7 (Ch7 p15.2)[55]. It has been reported that the expression of miR-196b is lower in CML patients than in healthy individuals. Recently, a study demonstrated that low level of expression of the tumor-suppressor, miR-196b can cause up-regulation of BCR-ABL1 expression which leads to the development of CML [55]. The dual luciferase reporter assay system also showed that *BCR-ABL1* is the target genes of miR-196b. Furthermore, the study reported that a decline in the expression of miRNA-196b, in the cells overexpressing it, can restore BCR-ABL1 protein levels, enhance cell multiplication, and impeded the synthesis (S) phase of the cell cycle. In addition, down-regulation of *BCR-ABL1* gene by small interfering (si) RNAs reduced the BCR-ABL1 protein levels and obstructed proliferation, similar to what was observed in cells displaying over-expression of miRNA-196b and a retarded G1 stage. It was also suggested that as regulation of miRNA-196b by DNA methylation is known to be involved in the progress of many other cancers it may hold true even for CML [55].

The miR-30a, generated from an intronic transcriptional unit, is located on human chromosome 6 (Ch6 q13) and belongs to miR-30 family [56, 57]. The miRNAs of miR-30 family has been found to be highly expressed in cardiac cells [58]. In an investigation, bone marrow samples of 16 CML patients and 10 normal patients, collected for the diagnosis of CML because of clinical features (hematological characters and presence of Philadelphia Chromosome), demonstrated that the expression of miR-30a is lower in bone marrow from CML patients than in normal control subjects [59]. It was also revealed that overexpression of the miR-30a in K562 cells (human immortalized myelogenous leukemia line) decreases the BCR-ABL1 protein levels, reduces cell proliferation and arrests the cells between G1 and S phase of the cell cycle. In contrast, inhibiting the expression of miR-30a in these cells notably increased the BCR-ABL1 protein levels, cell proliferation and restores the cell cycle. Furthermore, functional genomics studies in K562 cells verified that miR-30a played a tumor suppression role in CML by regulating the expression of BCR-ABL1 [59]. In another study, it was suggested that imatinib considerably inhibits expression of miR-30a in human CML cells. In contrast, reduction of miR-30a by antagomiR-30a surges the expression of Beclin 1 and Autophagy protein 5 (ATG5), and inhibits imatinib-induced cytotoxicity [60].

The miR-29 is a family of small RNA molecule in the shape of a stem-loop or hairpin. The miR-29 family in human includes hsa-miR-29a, hsa-miR-29b-1, hsa-miR-29b-2, and hsa-miR-29c. miR-29b-1 and miR-29b-2 have identical mature sequences, which are together called miR-29b [61, 62]. Mature miR-29s are highly conserved in rat, mouse and human, and share identical seed sequences at 2 to 7 nucleotide positions [63]. The genes coding for the precursors of miR-29b-1 and miR-29b-2 are located on chromosome 7 (Ch7 q32.3) [33] and chromosome 1 (Ch1 q32.2), respectively [64]. A recent study utilizing luciferase reporter assay demonstrated that miR-29b considerably reduces the activity of a luciferase reporter containing ABL1-3′UTR [65]. Another investigation showed that forced expression of miR-29b in K562 cells inhibits cell growth, colony formation ability and induces apoptosis through cleavage of procaspase 3 and Poly ADP ribose polymerase (PARP). Which suggests that miR-29b induced reduction of BCR-ABL1 protein in K562 cells is sufficient to trigger apoptotic response [66]. Thus, a prominent reduction of miR-29b in CML might implicate miR-29b as a potential tumor suppressor in CML by targeting ABL1 and BCR/ABL1 [67]. It had also been documented that miR-29b could impact CML cell proliferation and induces apoptosis via regulation of BCR/ABL1 protein and Ribonuclease latent (RNase-L) [68]. The microarray studies of miRNAs downregulated in CML blast crisis revealed that miR-29b expression was significantly lower in CML patient samples as compared with normal volunteers [39]. In CML, abnormal expression of miR-29 family has been described [69] and a recently performed qPCR analysis of miR-29b expression further suggested that miR-29b was significantly downregulated in CML patient samples, suggesting that miR-29b negatively regulates ABL1 and BCR/ABL1, post transcriptionally [70, 71].

The miR-138 family was first detected in humans (*Homo sapiens*) [72]. miR-138 is usually considered as an example of the post-transcriptional control of miRNAs. Precursor form of miR-138 (pre-miR-138-2) is ubiquitously expressed in all tissues but, the mature miR-138 is spatially restricted to only certain tissue and cell types. It was observed that pre-miR-138-2 is cleaved to its mature form by Dicer in nucleus and is exported to cytoplasm only in distinct cells [73]. In the human genome, there are two miR-138 associated genes which are not located in any cluster. In particular, the miR-138-1 and miR-138-2 gene is located on chromosome 3 (Ch3p21.32) and chromosome 16 (Ch16q13), respectively [74]. In a current study, it was revealed that miR-138 binds to the coding region of ABL protein instead of 3′UTR of ABL mRNA. However, this binding downregulated the expression levels of ABL and BCR-ABL proteins which then causes inhibition of cell proliferation. Moreover, study demonstrated that the expression of miR-138 is triggered by treatment of imatinib which enhances the activity of GATA-binding factor 1(GATA1) and promotes its binding to miR-138 promoter. Generally, this expression of miR-138 is repressed by BCR-ABL. Therefore, miR-138, by the advantage of a BCR-ABL/GATA1/miR-138 integrated circuitry, acts as a tumor suppressor miRNA involved in the pathogenesis of CML and its clinical response to imatinib. The tumor suppressor activity of miR-138 was demonstrated in K562 and Ku812 cells, over expressing miR-138, by the initiation of G0/G1 cell cycle arrest, inhibition of cell proliferation and enhanced imatinib-induced apoptosis. Though de-regulated expression of this miRNA has been documented in a diverse array of tumors, it was revealed that miR-138 expression is down regulated in K562 cells and primary CML samples, which can be restored after imatinib treatment. Moreover, overexpression of miR-138 leads to the down regulation of BCR-ABL suggesting that there is negative regulatory loop between miR-138 and BCR-ABL. [75].

#### CRK family proteins

The first member of CRK family (v-CRK) of adaptor proteins was detected in late 1980s as the oncogene fusion product of the avian sarcoma virus, CT10 [76]. The CRK family is known to comprise of five members namely, v-CRK, CRKI, CRKII, CRKIII and CRK-like protein (CRKL) [77]. The cellular homolog of v-CRK were found to have an SH2 domain and either one (for CRKI) or two (for CRKII) SH3 domains [78]. These domains (SH2 and SH3) bind to phosphorylated tyrosine and proline-rich motifs, respectively [79]. CRKIII is predicted to encode a protein which have truncated C-terminal SH3 domain [80]. CRKL shares overall 60% of homology with CRKII, with one SH2 and two SH3 domains [81]. In adult murine tissues, CRKL expression is highest in adult hematopoietic tissues and lower in many epithelial tissues, whereas CRK displays elevated expression in the brain, lung, and kidney while exhibits low expression in bone marrow [79]. CRK proteins are prevalent phosphorylation substrates for the *BCR-ABL* fusion oncogene and are found in more than 95% of CML and 20% to 30% of acute lymphoblastic leukemia cases. CRKL is a key tyrosine-phosphorylated protein present in neutrophils of CML patient [82]. The amino terminal end (N-) of CRKL (SH3N domain) binds precisely to a proline-rich region in the carboxyl (C-) terminus of BCR-ABL protein. CRKL was also determined as a constitutively phosphorylated 39 kD tyrosine phosphoprotein in CML cells [83]. Guanine nucleotide exchange factor (GEF) or SOS1 protein is a major CRK SH3N- domain binding protein, which causes the activation of RAS and allow CRK to couple diversified upstream signals [80, 84].

miRNAs play an essential role in regulating CRK and CRKL. miR-126 is located within the 7th intron of the *EGFL7* gene, residing on human chromosome 9 (9q34.3) [85] while, miR-17 is positioned on chromosome 13 (13q31.3) and belongs to the miR-17-92 cluster [86]. The putative target gene of these two miRNAs is CRK protein, which is involved in the MAPK signaling pathway. It was also suggested that miR-126 in humans is expressed only in endothelial cells, throughout capillaries as well as larger blood vessels [87], and acts upon various transcripts to control angiogenesis [85]. Recently, a study demonstrated that both, miR-126 and miR-17 were up-regulated in blast crisis (BC) samples of CML patients [88]. Study also suggested that miR-17 may be able to regulate MAPK expression level in leukocytes.

miR-221 is a tiny RNA molecule whose gene is located on the X chromosome (Xp11.3) [89]. This human miRNA was detected by a computational approach using conservation with mouse and *Fugu rubripes* sequences [90]. Expression of the excised miRNA were first validated in zebrafish, and later on in human promyelocytic leukemia cell 60 (HL-60) [91]. Recent study showed that the miR-221 level was up-regulated in BC samples of CML patients [88]. Research have demonstrated that miR-221 have an important role in the regulation of apoptosis by directly affecting the pro-apoptotic molecule, p53 upregulated modulator of apoptosis (PUMA), *in vitro* and xenograft mice model [92]. In a separate study, miR-128, miR-15a and miR-188-5p were suggested to suppress CML via CRKL encoding v-CRK avian sarcoma virus CT10 oncogene homolog-like [93]. The sequence of miR-188 was predicted based on homology to a documented miRNA from mouse [94]. This miRNA is located on the X chromosome (Xp11.23) [95]. Similarly, miR-128a sequence was also predicted based on homology to a verified miRNA from mouse [96]. Later on, the expression of miR-221 was even reported in HL-60 cells [91]. The miR-128a is located on chromosome 2 (2q21.3). The sequence of miR-15 was retitled as miR-15a, which can form a cluster with miR-16 within 0.5 kb at chromosome position 13 (13q14.2) and belongs to the miR-15 family [97]. It is found to be overwhelmed in chronic lymphocytic leukemia [98]. Information about the significance of miRNAs in the pathogenesis of CML is limited to the description of the abnormal expression of miR-15a in the CML cell line K562 [99].

#### SOS proteins

SOS specify to a set of genes that were first identified in *Drosophila melanogaster* as a functional gene product downstream of sevenless protein-tyrosine kinase in the RAS/MAP kinase pathway [100, 101]. It encodes GEF that acts upon the RAS subfamily of small GTPases. Mammalian cells possess two types of SOS homologs, SOS1 (~170 kDa) and SOS2 (~150 kDa), derived from divergent genetic loci. The N-terminal end of SOS1 encodes a Dbl homology (Dbl) and Pleckstrin homology (PH) duo that exchanges GTP for GDP on RAS. While, tandem C-terminal proline-rich motifs interacts with several adaptor proteins, including Grb2 and E3b1 (a Rac1 GEF) [102]. Both SOS1 and SOS2 carry repetitive proline motifs that conform to consensus CRK SH3 binding motifs and display a stable association between CRK and SOS.

Recently, it was predicted by TargetScan analysis that miR-155 is a putative target of SOS1 gene. This miRNA is processed from an exon of a noncoding RNA transcribed from the B-cell Integration Cluster (BIC), located intergenically on chromosome 21 (21q21.3), expressed in activated B cells, T cells, monocytes and macrophages [103]. BIC shows robust sequence homology amongst human, mouse and chicken and is highly, although not exclusively, expressed in lymphoid organs suggesting an evolutionary conserved function [96, 104, 105]. It was revealed from miRNA-based microarray and miR–quantitative Polymerase Chain Reaction (qPCR) analysis that the miR-155 is abnormally downregulated in K562 cells, in CML cell lines, and in patients with CML as compared to non-CML cell lines and blood samples from healthy patients [103].

miRNA-181a (miR-181a), one of the copious miRNAs conserved among vertebrates, is differentially expressed in a variety of leukemia. The miR-181 family contains four immensely conserved mature members, i.e., miR-181a, miR-181b, miR-181c and miR-181d, which are derived separately from 6 precursors located on 3 different chromosomes [106]. The miR-181a-1 and miR-181b-1 are clustered together and located on chromosome 1, miR-181a-2 and miR-181b-2 are clustered together and located on chromosome 9 [107], and miR-181c and miR-181d are clustered together and located on chromosome 19 [108, 109]. It had been found that the expression of miR-181a is very low, so low that it cannot be detected by qPCR in K562 cells, indicating that downregulation of miR-181a might play a major role in leukemogenesis. The combination of TargetScan and miRNAmap software prediction proposed that SOS1 is the putative target genes of miR-181a. Moreover, it also revealed that the expression pattern of miR-181a was deregulated in CML patients [110].

#### K-RAS proteins

RAS proteins are small GTPases. These proteins are known to be preserved across species and play key roles in numerous basic cellular functions, including control of proliferation, cell growth differentiation, and apoptosis [111]. RAS proteins acts as molecular switches that cycle between two conformational states: one when they are bound to GTP (the active form) and another one when bound to GDP (the inactive form) [112–115]. GEFs promote the formation of GTP-bound RAS [116] whereas, GTPase-activating proteins, or GAPs, stimulate the hydrolysis of GTP on RAS, returning them to their inactive state [117]. Transgenic and cell-biological studies [118–121] complemented by clinical observations [122] actively pointed out that RAS has different iso-forms i.e., H-RAS, N-RAS and K-RAS. These isoforms can provoke distinct signal outputs, although interacting with a prevalent set of activators and effectors. This biological disparity is probably considered due to the C-terminal amino acids (25 in numbers) of the Hypervariable domain (Hvr), which is the only region that differs significantly [111]. Physiological and oncogenic activation of RAS trigger a broad range of downstream signaling pathways, of which, the RAF-MEK-ERK pathway was the first to be identified as RAS effector pathway [123–126].

Bioinformatics analysis utilizing TargetScan revealed that KRAS is the probable target of miR-155 and miR-19a. As discussed earlier, miR-155 is encoded from the gene located on miRNA chromosome 21 (21q21.3) [127, 128]. In humans, this miRNA is transcribed from the *MIR155* parent gene or *MIR155HG* [129, 130] and the MIR155HG RNA transcript does not have a long open reading frame (ORF), however, it does consists of an imperfectly base-paired stem loop that is conserved across species. Subsequent studies established that the *MIR155HG* was composed of three exons that span a 13 kb region [131]. In a study Rokah et al. utilized miRNA microarray to identify miRNA expression in CML cell lines and patient samples. Among several dysregulated miRNAs identified in CML cell lines, expression levels of miR-155 was too found downregulated [103]. A decrease in miR-155 may establish an additional mechanism leading to deregulated expression of RAS. From PCR analysis, K-RAS has been shown to cooperate with ABL to induce full *in-vitro* and *in-vivo* transformation of cells leading to tumorigenesis. Moreover, it was found that the expression level of K-RAS was significantly higher in K562 cells compared to normal blood samples [103]. Downregulated expression of miR-155 and higher levels of K-RAS in CML patients points toward the possible regulation of K-RAS by miR-155. The potential of miR-155 in regulating K-RAS may be a unique pathway of regulation that can be targeted for therapeutic purpose and hence, requires further researches for determining the possibilities.

miR-19 is the key oncogenic component of the *miR-17-92* cluster, and, therefore, miR-19-specific targets are likely to mediate the oncogenic effects of the *miR-17-92 cluster* (also known as oncomiR-1) [132]. The *miR-17-92* miRNA cluster produces a single polycistronic primary transcript that yields six mature miRNAs: miR-17, miR-18a, miR-19a, miR-20a, miR-19b, and miR-92a. This distinctive structural feature of *miR-17-92*, common in vast number of miRNA genes in mammalian genomes, may be responsible for the molecular basis for its pleiotropic functions in a cell type-dependent and context-dependent manner [133]. *miR-19a* is located on human chromosome 13 (13q31.3). Targetscan analysis identified that, K-RAS, which is involved in MAPK signaling, is a predicted target of miR-19a. Abnormal expression of onco-miR, miR-19a was described in CML CD34^+^ cells. Study further revealed that the level of miR-19a is up-regulated in the CML cell line and it may act as oncomiRs [134].

#### RAF1 protein

The cytoplasmic protein, RAF1 has been verified as a key molecule in MAPK signaling pathway. RAS subfamily of membrane associated GTPases are known to activate downstream RAF1 proteins [135]. In humans it is encoded by the *RAF1* gene [136]. Therefore, RAF1 is an interesting target for molecular therapies and target-based therapies which are widely considered to be the future of cancer treatment. The *RAF1* gene is positioned on the short (p) arm of human chromosome 3 at position 25 (3p25) [137]. As discussed in above section, miR-19a is located on human chromosome 13 and belongs to the miR-19 family of the *miR-17-92 cluster*. It was recently demonstrated that the expression pattern of miR-19a was found up-regulated in CML patients [133]. The confirmed increase of miR-19a was also identified in samples of BC pool [134]. In addition, TargetScan analysis identified RAF1 as a probable target of the oncogenic miR-19a. However, future studies are needed to authenticate the role miR-19 in CML via regulation of RAF-1.

#### MAPK/ERK protein

MAPKs are eukaryotic protein Ser/Thr kinases, which is encoded by *MAPK1* gene and activated by an upstream activator, RAF, in the MAPK signaling pathway [5]. The conventional MAP kinases can be assembled into three major families. These are ERKs (extracellular-signal-regulated kinases), JNKs (Jun amino-terminal kinases), and p38/SAPKs (stress-activated protein kinases) [138]. All MAPKs comprise a Serine/Threonine kinase domain flanked by N- and C-terminal regions of diverse lengths. Different additional domains also exists in some MAPKs, comprising a transactivation domain (TAD), a region conserved in ERK3 and ERK4 (C34), a nuclear localization sequence (NLS), and a domain rich in alanine, histidine, and glutamine (AHQr) [5]. It is well known that regulation of both RAS and RAF is crucial for the proper maintenance of cell proliferation, as activating mutations in these genes lead to oncogenesis [1, 139]. Activated RAF binds to and phosphorylates the dual specificity kinases MEK1/2, which in turn, phosphorylate ERK1/2 within a conserved Thr-Glu-Tyr (TEY) motif in their activation loop [5, 32, 136].

Several miRNAs play regulatory role in MAPK1 expression, of which, miR-17 and miR-19a can directly regulate the MAPK1. miR-17 and miR-19a belongs to miR-17 and miR-19 miRNA family, respectively and are members of highly conserved miR-17-92 cluster in vertebrates [140]. Both of these miRNAs are located on human chromosome 13 (13q31.3). The 17-92 miRNA clusters is recognized as an important CML-associated oncogene. An investigation reported that overexpression of this cluster stimulates cell proliferation in K562 cell line [141]. Moreover, this cluster is important for cell cycle, apoptosis and other pivotal processes. Often miR-17-92 cluster is found dysregylated in hematopoietic and solid tumors. A study also reported that miR-17 is expressed from the 3′ arm of the hairpin precursor in human epithelial carcinoma cell line (HeLa) cells [97]. Interestingly, it was described that RAS/MAPK signaling may contribute to the survival of BCR-ABL^+^ cells under imatinib selection pressure [142]. PCR analysis has demonstrated that the levels of miR-17 and miR-19a are up-regulated in CML patients as compared to non-CML patients [88]. Another study dealing with miRNA expression in CML demonstrated abnormal expression of the miR-17 and miR-19a in CML CD34^+^ cells [134].

### Future direction

CML has appeared as one of the significant challenges for the scientific community who are dealing with cancer and new drug discovery. Over the last few years, several interesting studies have been performed on the regulatory mechanism of CML and its pathogenesis. Recently, studies are paying attention on the identification of small miRNAs and there contribution toward regulating key events of the cells. Current review will help to understand the contribution of these recently identified miRNAs in the regulatory complexities of CML. Our comprehensive study will also help the researchers to target and verify the novel miRNA-based diagnostics for this leukemia. Furthermore, these miRNAs may be utilized as therapeutics to upregulate or downregulate the differentially expressed proteins related to MAPK pathways in CML in upcoming time.

## CONCLUSIONS

**Figure 2:**
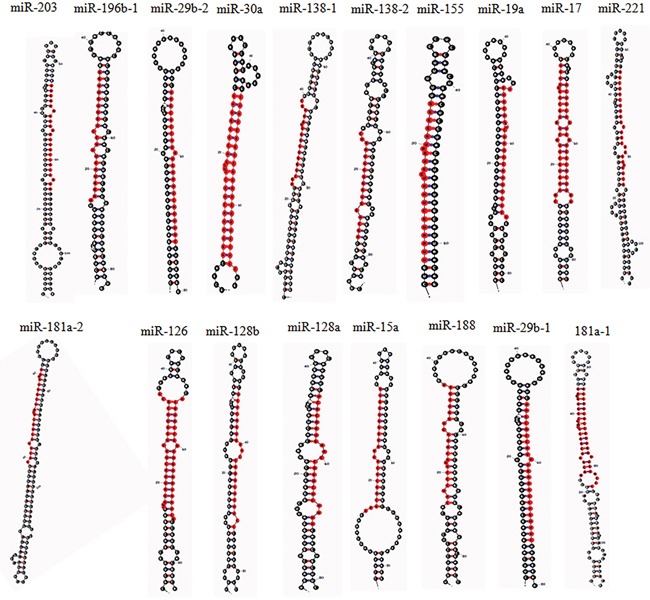
The Stem-loop structure of human miRNAs related to MAPK signaling pathway in CML, (determined by miRNAMAP; http://mirnamap.mbc.nctu.edu.tw/) [143]

Growing evidences indicates the significance of miRNAs in modulating signal transduction pathways in CML (Figure 1). With the aid of computational tools, we have summarized a number of miRNAs that are being identified to regulate proteins cascades during CML (Figure 2). Our study has highlighted the role of miRNAs in CML and at the same time has explored new possibilities in the field of leukemia. In summary, we have depicted an extensive connection between miRNA expression and human CML which shows the dual functions of miRNAs as oncogene and tumor suppressor. We can conclude that the ability of miRNAs to control different cellular processes in various tissues at multiple levels makes them one of the most competent therapeutic agents in modern medicine. However, more fundamental understanding of miRNA regulated MAPK signal transduction pathways is required to address deeper insight into the mechanism of CML which can help to develop novel miRNA/anti-miRNA-based therapeutics in the near future.

## Abbreviations

BCR-ABL: breakpoint cluster region- Abelson murine leukemia viral oncogene homolog, non-receptor tyrosine kinase
CRK: chicken tumour virus no. 10 [CT10] regulator of kinase
CRKL: v-crk avian sarcoma virus CT10 oncogene homolog-like
KRAS: v-Ki-ras2 Kirsten rat sarcoma viral oncogene homolog
NRAS: neuroblastoma RAS viral (v-ras) oncogene homolog
HRAS: Harvey rat sarcoma viral oncogene homolog
RAF1: Raf-1 proto-oncogene, serine/threonine kinase
SOS1,2: son of sevenless homolog 1 (Drosophila), 2
Mek1,2: mitogen-activated protein/extracellular signal-regulated kinase kinase 1, 2
ERK: extracellular signal-regulated kinase
v-Crk: V-Crk Avian Sarcoma Virus CT10 Oncogene Homolog
EGFL7: Multiple Epidermal Growth Factor-Like Domains Protein 7
GTPase: guanosine triphosphatase.
